# Chemokine receptor 4 expression is correlated with the occurrence and prognosis of gastric cancer

**DOI:** 10.1002/2211-5463.12864

**Published:** 2020-05-07

**Authors:** Yang Li, Hong‐Chang Wang, Jin‐Shen Wang, Bo Sun, Le‐Ping Li

**Affiliations:** ^1^ Department of Gastrointestinal Shandong provincial hospital affiliated to Shandong university Jinan China

**Keywords:** CXCR4, CYT, gastric cancer, immune infiltration, TMB, tumor purity

## Abstract

Gastric cancer (GC) is a common tumor with a low 5‐year survival rate. The chemokine receptor 4 (CXCR4) protein contributes to the progression and prognosis of GC, but the relationship between CXCR4 and immune infiltration, somatic copy number alteration (SCNA), tumor purity, tumor mutation burden (TMB), cytolytic activity (CYT), and drug sensitivity in GC is poorly understood. This study aimed to systematically explore the role of CXCR4 in GC. Microarray and RNA‐seq data were collected from the Gene Expression Omnibus and The Cancer Genome Atlas. Our analysis shows that CXCR4 is correlated with various types of immune cells. Patients with high CXCR4 expression had a higher fraction of B cells and CD8^+^ T cells, and a lower fraction of CD4^+^ T cells. In addition, high CXCR4 expression was associated with more advanced tumor stage, worse prognosis and higher stromal score, immune score, and cytolytic activity (*P* < 0.05). High CXCR4 expression also correlated with lower tumor purity and TMB. In summary, our analyses suggest that CXCR4 may affect the progression and prognosis of GC by influencing immune infiltration, TMB, CYT, tumor purity, and drug sensitivity.

## Graphical abstract

CXCR4 is a chemokine receptor, which regulates tumor growth, proliferation, and metastasis in cancers. We found that gastric cancer patients with overexpression of CXCR4 had lower TMB, purity, higher CYT, and CD8 T‐cell infiltration. The patients also exhibited reduced sensitivity to 17‐AGG, trametinib, and docetaxel. Collectively, these findings suggest that patients with overexpression of CXCR4 have worse prognosis.

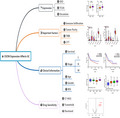

**Abbreviations**
CXCR4: chemokine receptor 4
CYT: cytolytic activity
EMT: epithelial‐to‐mesenchymal transition
GC: gastric cancer
GO‐BP: gene ontology biological process
SCNA: somatic copy number alteration
TMB: tumor mutation burden

Gastric cancer (GC) is one of the top three most lethal cancers in the world [1]. Despite the improvements in surgery and the development of various medical detection technologies, its 5‐year survival rate is still very low [2, 3]. In recent years, the bioinformatics approach has become a research hotspot to investigate the occurrence, progression, and treatment of cancer [4].

Chemokine receptor 4 (CXCR4) is a chemokine receptor that has been found to be overexpressed in various types of cancer, including leukemia, breast cancer, and prostate cancer [5, 6]. Moreover, its contribution to regulating tumor growth, proliferation, and metastasis in various types of cancers has been reported [7, 8, 9]. In GC, CXCR4 can regulate the tumor epithelial‐to‐mesenchymal transition (EMT) and the progression through the PI3K/AKT pathway, or induce EMT through the cross talk with c‐MET signaling. It can also influence the proliferation and invasion process through the Wnt/β‐Catenin pathway [6, 10, 11, 12, 13, 14]. Hence, CXCR4 can help regulate the development and prognosis of GC.

In recent years, researchers have focused their attention on different cancer parameters, including immune infiltration, tumor mutation burden (TMB), somatic copy number alterations (SCNA), tumor purity, cytolytic activity (CYT), and drug sensitivity [15, 16, 17, 18, 19, 20]. These parameters have all been reported to be prognostic factors and potential therapeutic markers for various cancers. However, to the best of our knowledge, there are no studies investigating whether the expression of CXCR4 is linked to these parameters, and how this protein can affect the progression and prognosis of GC. The present study aimed to fill this knowledge gap. We used available GC patient data from the Gene Expression Omnibus (GEO) and The Cancer Genome Atlas (TCGA), and investigated the role of CXCR4 in GC in relation to the clinical characteristics, tumor purity, immune infiltration, TMB, CYT, survival, and other parameters.

## Materials and methods

### Data collection

Primary observation was performed using the Oncomine database (https://www.oncomine.org/) and TIMER (https://cistrome.shinyapps.io/timer/). We performed a systematic search in the GEO (https://www.ncbi.nlm.nih.gov/geo/) and TCGA (https://cancergenome.nih.gov/) databases. Four data sets on the GEO database with relevant clinical information were considered as cohort 1 (combined *n* = 726): http://www.ncbi.nlm.nih.gov/geo/query/acc.cgi?acc=GSE66229 [21] (*n* = 400), http://www.ncbi.nlm.nih.gov/geo/query/acc.cgi?acc=GSE15459 [22] (*n* = 200), http://www.ncbi.nlm.nih.gov/geo/query/acc.cgi?acc=GSE57303 [23] (*n* = 70), and http://www.ncbi.nlm.nih.gov/geo/query/acc.cgi?acc=GSE34942 [24] (*n* = 56). In total, 413 GC samples from TCGA were selected as cohort 2. Microarray expression profiles were obtained using Affymetrix Human Genome 133 plus 2.0 Gene Chips. The Batch function was used to consolidate the four data sets. Data normalization was performed, and gene expression levels were computed as mean values of all annotated probe sets [25]. edger was used for the differential gene analysis of RNA sequencing data from TCGA.

### Analysis of correlation between CXCR4 expression and basic clinical characteristics

According to the median expression of CXCR4, we divided patients into two groups: a high CXCR4 expression group (CXCR4‐H) and a low CXCR4 expression group (CXCR4‐L). Differentially expressed genes (*P* < 0.05, |log(FC)| ≥ 1) were identified between the two groups and analyzed using Gene Ontology (GO) and Kyoto Encyclopedia of Genes and Genomes (KEGG) by david (https://david.ncifcrf.gov/) [26]. The following basic clinical characteristics were collected for comparison: patient age, gender, tumor stage, microsatellite instability (MSI), and overall survival (OS).

### Analysis of the correlation between CXCR4 expression and immune infiltration, TMB, CYT, SCNA, and drug sensitivity

We calculated the immune infiltration, SCNA, tumor purity, TMB, CYT, and drug sensitivity to identify the correlation between CXCR4 expression. The results with *P* < 0.05 were considered statistically significant. The TIMER Web site was used for the primary analysis of the correlation between CXCR4 and immune cells, and SCNA of the CXCR4 gene. Then, we performed a Spearman's correlation analysis of the immune cell markers using TIMER, and the immune genes were selected according to previously published methodology [20, 27]. The cibersort (https://cibersort.stanford.edu/) method and LM6 gene signature were used for immune infiltration analysis between the CXCR4 expression groups [28]. The CYT of each patient was calculated based on transcript levels of two key cytolytic effectors, granzyme A (GZMA) and perforin (PRF1) [20]. The TMB of TCGA patients was calculated from the Genomic Data Common (GDC) data portal. The ESTIMATE package was used to calculate the tumor purity, stromal score, and immune score [29]. Data from Genomics of Drug Sensitivity in Cancer (GDSC) (https://www.cancerrxgene.org/) were obtained for the analysis of the effect of CXCR4 on drug sensitivity [30]. The Pearson's correlation was calculated between CXCR4 expression and drug half maximal inhibitory concentration (IC50). The results with a threshold of *P* < 0.01 were considered statistically significant.

### Statistical analysis

All statistical analyses were conducted using r (The R Foundation, Vienna, Austria) and the spss version 23.0 software (IBM, Armonk, NY, USA). Graphical representations were generated using the graphpad prism 7 software (GraphPad Software, San Diego, CA, USA). Chi‐square and Wilcoxon rank‐sum tests were used for categorical and continuous variables, respectively. Student's *t*‐test was used for data with normal distribution and homogeneous variance.

## Results

### Differential gene expression

**Fig. 1:**
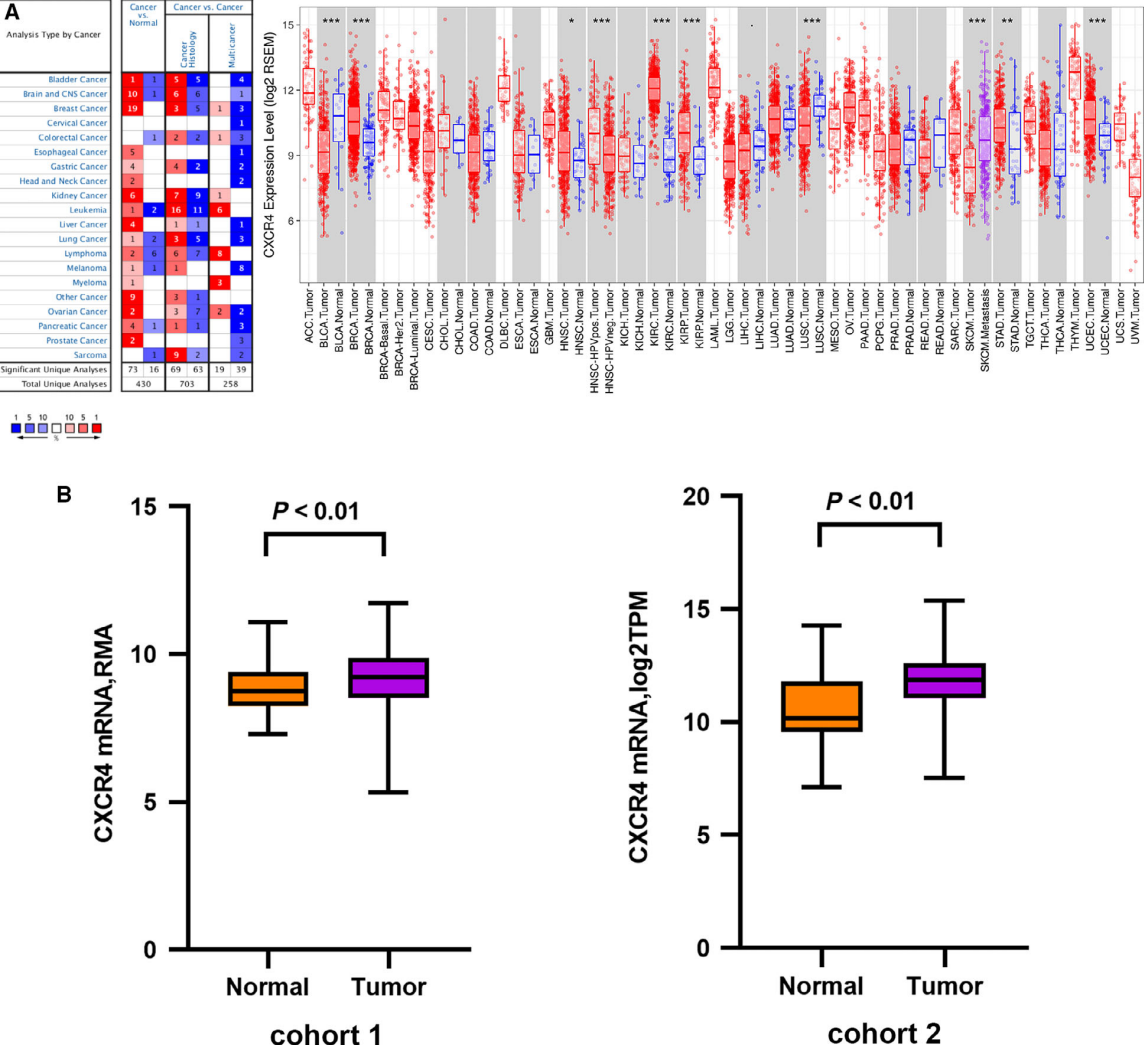
Differential CXCR4 expression. (A) The differential expression of CXCR4 from Oncomine and TIMER; (B) The differential *CXCR4* expression in samples from cohort 1 and cohort 2. Data are represented as mean ± SD.

By using Oncomine and TIMER, we found that CXCR4 expression was higher in GC patients compared with healthy controls. Patients from the GEO were considered as cohort 1, and those from the TCGA were considered as cohort 2. This result was consistent in both databases (Fig. 1A,B).

**Fig. 2:**
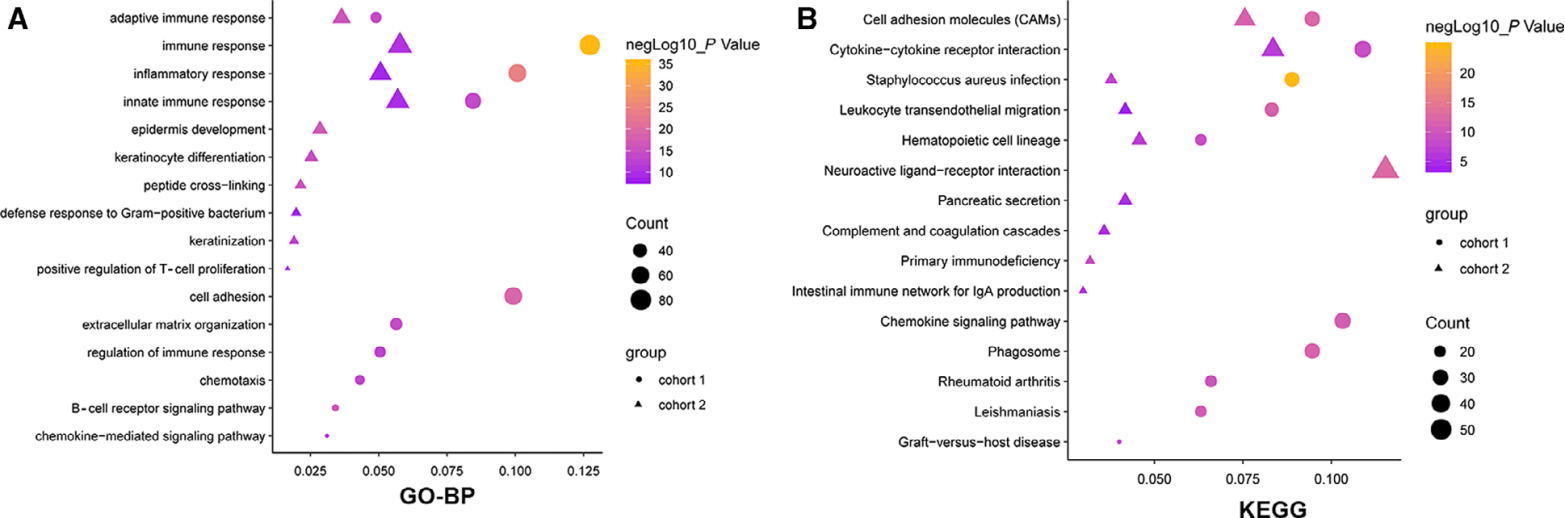
GO‐BP (top) and KEGG (bottom) analysis. The BP (left) and KEGG (right) enrichment of the differential genes between the *CXCR4*‐H and *CXCR4*‐L groups in two cohorts. Circle represents cohort 1, and triangle represents cohort 2. The size of the symbol indicates the gene count; the color indicates the −Log10 of the *P* value evaluating the statistical significance of the relative enrichment.

We divided the samples into two groups based on the CXCR4 expression levels, high (CXCR4‐H) and low (CXCR4‐L). We found 778 and 1552 differentially expressed genes between the groups in the training and in cohort 2, respectively. We identified the genes by performing a Gene Ontology Biological Process (GO‐BP) and KEGG analysis (Fig. 2, Table S1). The GO‐BP analysis results showed that the differentially expressed genes were mainly involved in the regulation of the immune response and the inflammatory response. The KEGG results showed that they were mainly involved in migration and adhesion. These biological functions and signaling pathways are closely related to the development of GC.

### Relationships with clinical characteristics

**Fig. 3:**
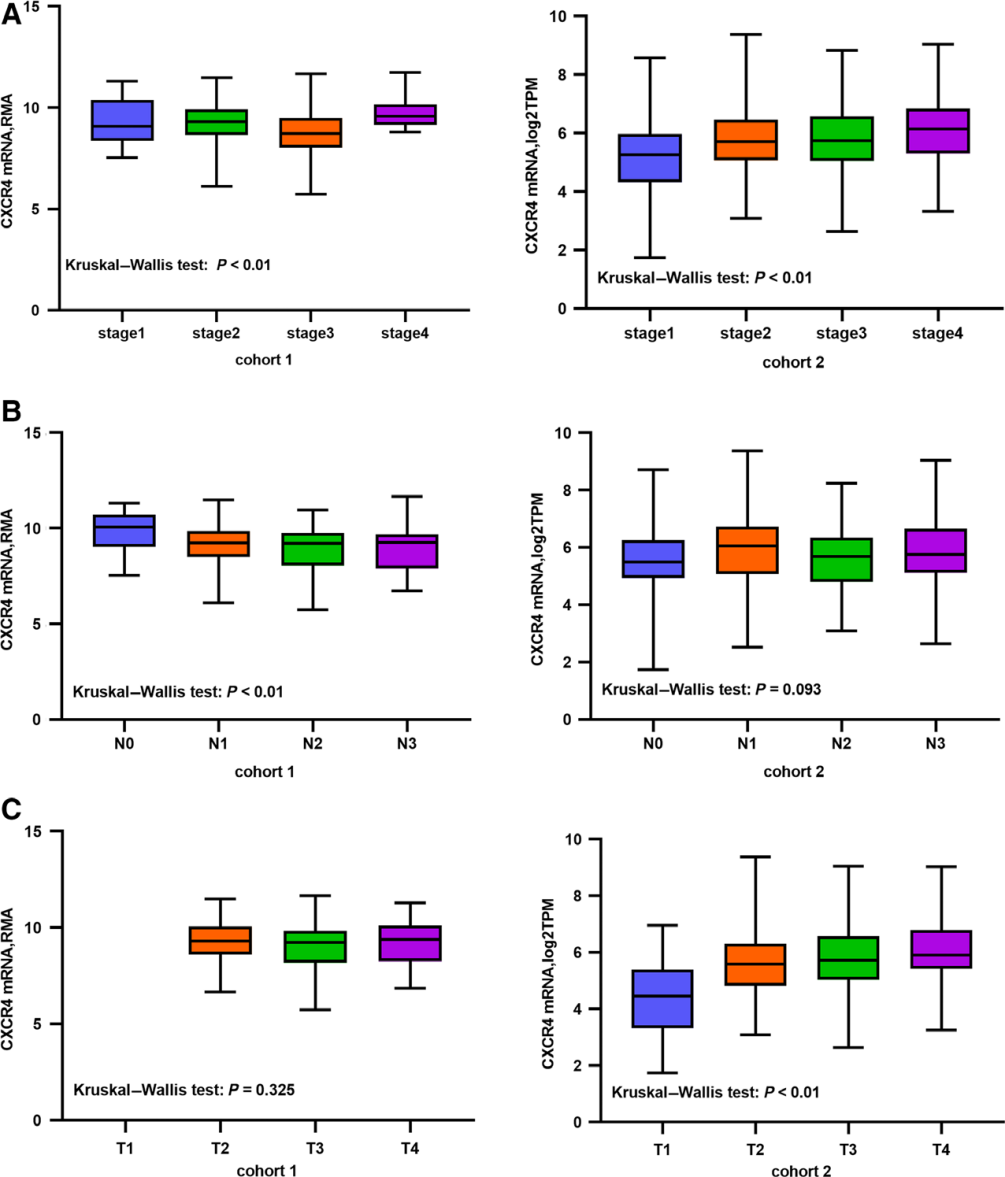
The CXCR4 expression in clinical TNM stage. (A) *CXCR4* expression in different stages. (B) *CXCR4* expression in different N stages. (C) *CXCR4* expression in different T stages. Data are represented as the mean ± SD.

We then performed a correlation investigation in both cohorts between the CXCR4 expression and various clinical characteristics (Table 1). For the purpose of comparison, we classified clinical stage I and II tumors as early stage and stage III and IV tumors as advanced stage. A similar criterion was used for T and N stage. We found a significant difference between the groups in relation to the clinical stage both in the training and in cohort 2. The CXCR4‐H group had more patients with advanced stage tumors compared with the CXCR4‐L group in both cohorts (*P* < 0.05), and the expression of CXCR4 gradually increased with the increase in stage, especially in cohort 2 (Fig. 3A). In cohort 1, we also found a statistical difference between the groups regarding age, gender, N stage, and MSI (*P* < 0.05). No significant difference could be found for N stage (Fig. 3B). In cohort 2, we found a significant difference for the T stage (*P* < 0.05). We also found a correlation between earlier T stages and the CXCR4‐L group (Fig. 3C). No difference in the M stages was observed in the two cohorts.

**Table 1:** Clinical characteristics. The relationship between CXCR4 expression and clinical characteristics in both training and cohort 2. MSS, microsatellite stability; MSI, microsatellite instability.

Group	Cohort 1			Cohort 2		
	CXCR4‐H	CXCR4‐L	*P*	CXCR4‐H	CXCR4‐L	*P*
Age (years)	*n* = 306	*n* = 306	<0.0001*	*n* = 185	*n* = 186	0.1642
≥ 60	179	236		123	136	
< 60	127	70		62	50	
Gender	*n* = 306	*n* = 306	0.0396*	*n* = 187	*n* = 188	0.6387
F	114	90		69	65	
M	192	216		118	123	
Stage	*n* = 306	*n* = 306	0.0176*	*n* = 158	*n* = 169	0.0155*
1	34	41		14	35	
2	58	87		52	55	
3	121	106		71	65	
4	93	72		21	14	
1 + 2/3 + 4	92/214	128/178	0.0024*	66/92	90/79	0.0378*
T	*n* = 191	*n* = 175	0.4687	*n* = 161	*n* = 172	<0.0001*
T1	0	0		1	18	
T2	102	92		32	40	
T3	71	72		71	86	
T4	18	11		57	28	
T1 + 2/T3 + 4	102/89	92/83	0.8735	33/128	58/114	0.0068*
N	*n* = 191	*n* = 175	0.0267*	*n* = 158	*n* = 165	0.2196
N0	36	14		41	59	
N1	76	80		49	39	
N2	51	54		35	36	
N3	28	27		34	31	
N0 + 1/ N2 + 3	112/79	94/81	0.3428	90/69	98/67	0.6109
M	*n* = 191	*n* = 175	0.5298	*n* = 158	*n* = 166	0.2282
M0	175	157		144	157	
M1	16	18		14	9	
MSS/MSI	*n* = 150	*n* = 150	0.0009*	*n* = 97	*n* = 113	0.8915
MSS	104	128		80	94	
MSI	46	22		17	19	

* *P* < 0.05.

### CXCR4 is a predictor of poor OS in patients with GC

**Fig. 4:**
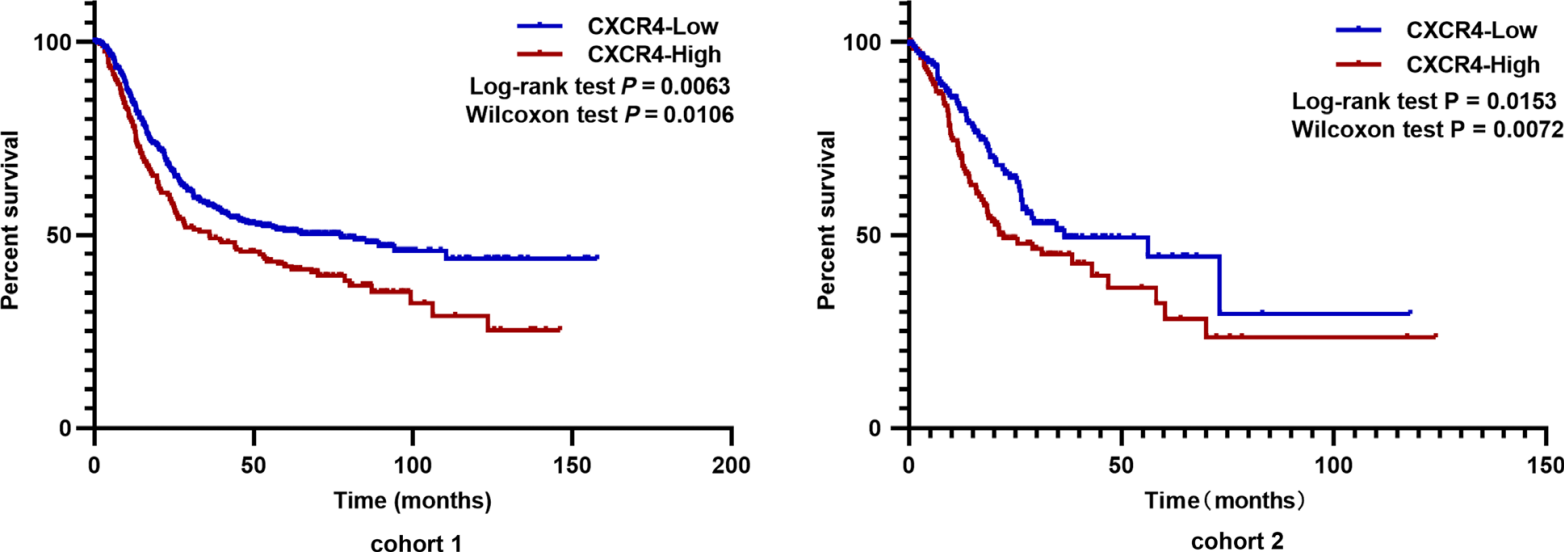
Comparison of the overall survival curve between the CXCR4‐H and CXCR4‐L groups. Both the log‐rank test and Wilcoxon test were performed for the significance comparison.

As shown in Fig. 4, we found a strong association between high CXCR4 expression and short OS in GC patients in both cohorts. Moreover, we found that CXCR4 expression had a significant effect on both the short‐term and long‐term survival of GC patients (Wilcoxon and Log‐rank test, *P* < 0.05).

### Comparison of immune cell type fractions

**Fig. 5:**
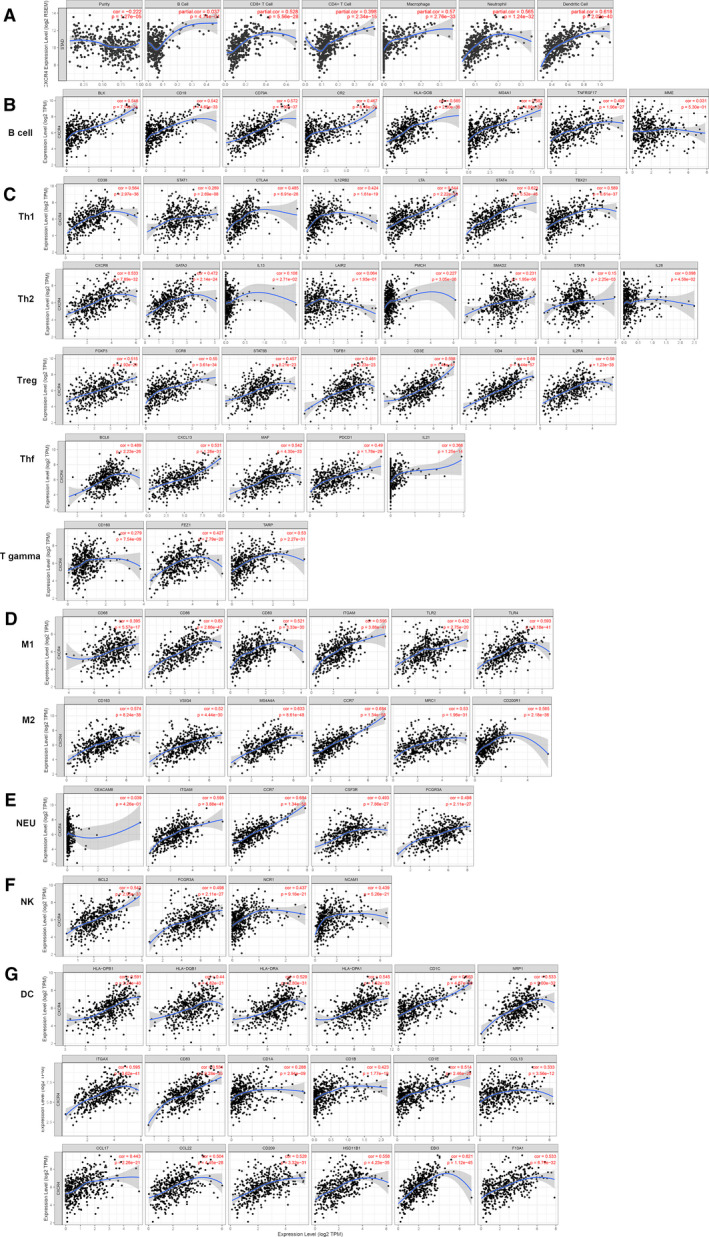
Correlation between CXCR4 expression and immune cells. (A) The correlation between *CXCR4* expression and six types of immune cells. (B–G) The validation of the correlation between *CXCR4* and gene markers of B‐cell and T‐cell subtypes (Th1, Th2, Tfh, Tregs, and T gamma), macrophages subtypes (M1 and M2), neutrophils, natural killer cells, and dendritic cells (Spearman's correlation).

By performing a TIMER analysis, we found a high correlation between CXCR4 expression and CD4^+^ T cells, CD8^+^ T cells, macrophages, neutrophils, and dendritic cells (Fig. 5A). Thus, we selected several related immune cell markers for further validation (Table S2). We observed a high correlation between CXCR4 expression and T‐cell subtypes (T‐helper type 1, T‐helper type 2, T follicular helper cells, regulatory T cells, and T gamma delta cells), B cells, macrophages subtypes (M1 and M2), neutrophils, natural killer cells, and dendritic cells (Fig. 5B–G). This result is consistent with our previous hypothesis that CXCR4 plays an important role in tumor immune infiltration in GC.

**Fig. 6:**
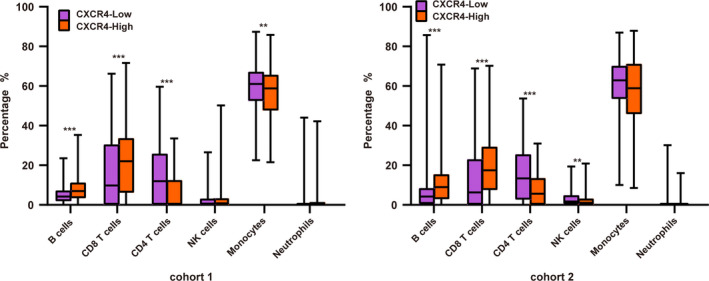
Comparison of 6 types of immune cells between the CXCR4‐H and CXCR4‐L groups. *P < 0.05, **P < 0.01, ***P < 0.005.

After CIBERSORT calculation, patients with a *P* value > 0.05 were removed, and nonparametric testing was performed to evaluate the statistical significance of the different immune cell fractions (Fig. 6). The results showed a significantly higher amount of B cells, CD4^+^ T cells, and CD8^+^ T cells (*P* < 0.001) in the CXCR4‐H group than in the CXCR4‐L group. In cohort 1, we observed fewer monocytes in the CXCR4‐H group (*P* < 0.05), while in cohort 2 we observed fewer NK cells (*P* < 0.05).

### Influence of CXCR4 expression on somatic copy number alterations in immune cells

**Fig. 7:**
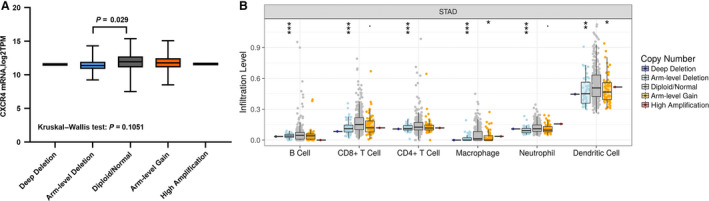
Immune infiltration levels with different somatic copy number alterations for the CXCR4 gene. (A) The mRNA level in different types. (B) The infiltration level for each SCNA category is compared with the normal using the two‐sided Wilcoxon rank‐sum test. ***P < 0.001, **P < 0.01, *P < 0.05, .*P* < 0.1.

We compared the immune infiltration levels among tumors with the presence of different somatic copy number alterations for the CXCR4 gene, by performing a TIMER analysis. We observed that when the CXCR4 gene had an arm‐level deletion, the expression of CXCR4 was reduced (*P* < 0.05) and the proportion of all types of immune cells was significantly decreased. When an arm‐level gain appeared instead, the expression of CXCR4 was reduced without significance, and the proportion of macrophages and dendritic cells decreased slightly, while the other types showed no significant changes (Fig. 7).

### Tumor purity, CTY, and TMB comparison

**Fig. 8:**
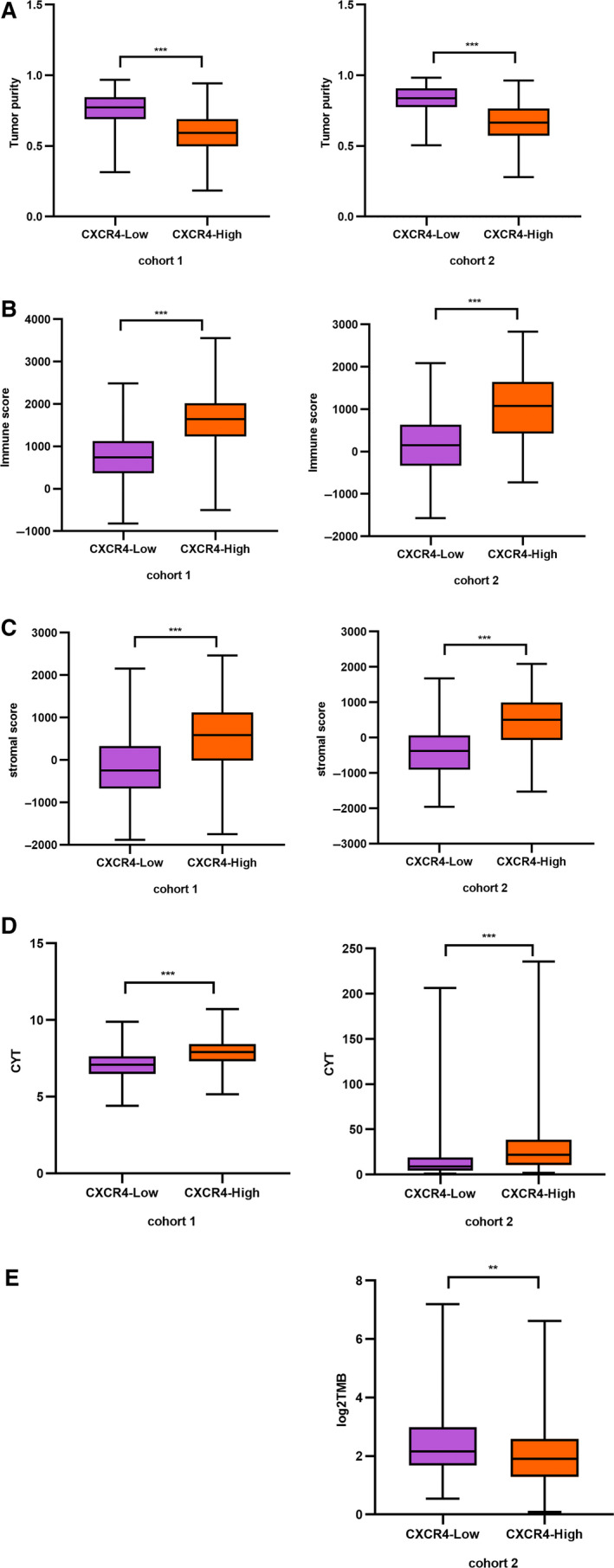
Tumor purity, CYT, and TMB comparison. (A) The comparison of the stromal score in cohort 1 (left) and cohort 2 (right); (B) the comparison of the immune score; (C) the comparison of the tumor purity; (D) the comparison of the CYT; (E) the comparison of log_2_TMB in the TCGA samples. Data are represented as the mean ± SD. *P < 0.05, **P < 0.01, ***P < 0.005.

The tumor purity, stromal score, and immune score were calculated by the ESTIMATE package, which inferred the level of infiltrating stromal and immune cells in tumor tissues and tumor purity using gene expression data [29]. We calculated the tumor purity value in the two cohorts. We found significant differences in tumor purity, immune score, and stromal score between the CXCR4‐H group and the CXCR4‐L group. The CXCR4‐H group had a higher stromal score and immune score, but a lower tumor purity (Fig. 8A–C). Both cohorts had similar results. Regarding the CYT activity, patients with high CXCR4 expression showed a stronger CYT activity in both cohorts (Fig. 8D). Regarding TMB, we converted the TMB count of the TCGA database into log2TMB, and the result showed that the TMB amount was lower in the CXCR4‐H group, indicating that the CXCR4‐H group may be less recognized and therefore less likely to be attacked by immune cells (Fig. 8E).

### Influence of CXCR4 on drug sensitivity

**Fig. 9:**
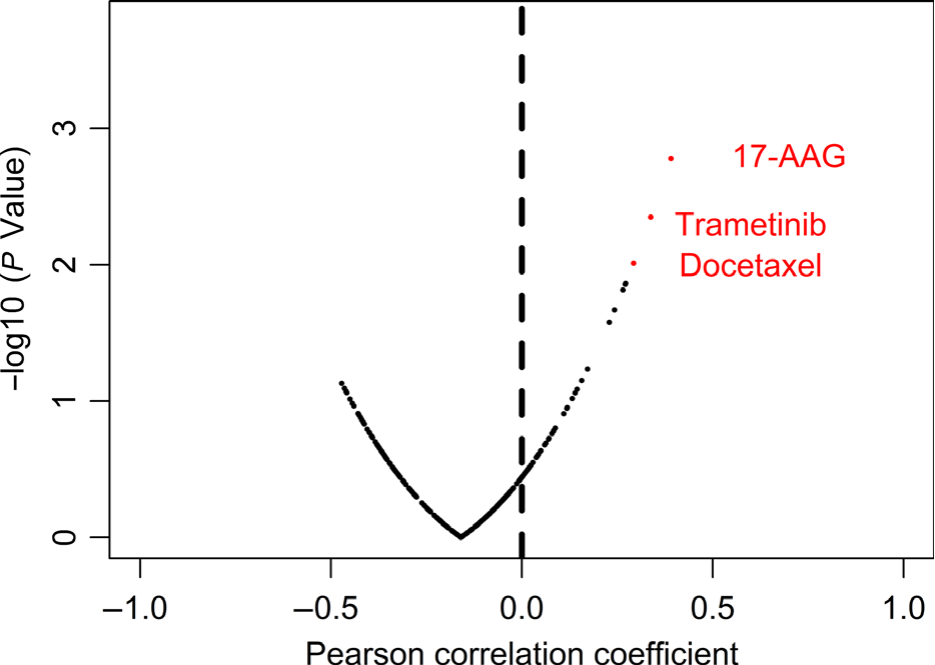
Correlation between drugs and CXCR4 expression. Pearson correlation was calculated, and red points are the drugs with a significantly increased resistance.

To analyze the effect of CXCR4 on drug sensitivity, we used data from the Genomics of Drug Sensitivity in Cancer database (GDSC). We found that three drugs, 17‐allylamino‐17‐demethoxygeldanamycin (17‐agg), trametinib, and docetaxel, which are used for GC treatment, met the screening criteria (*P* < 0.01) (Fig. 9, Table S3); their Pearson's correlation coefficients were 0.391, 0.338, and 0.293, respectively. The results showed that with the increase in CXCR4 expression, the drug IC50 is also increased.

## Discussion

Gastric cancer is a common malignant tumor characterized by a low 5‐year survival rate. In recent years, because of advances in chip and next‐generation sequencing technologies, more pathological parameters have been measured, such as TMB, CYT, immune infiltration, and tumor purity. These new parameters could potentially be used as therapeutic markers. Thus, numerous studies have focused on investigating their role in the development and prognosis of GC.

CXCR4 is a member of the C‐X‐C chemokine receptor family, which has been widely investigated in many cancers. It can regulate the biological behavior of GC cells through multiple pathways, such as the classical AKT pathway, or the alternative MAPK and Wnt pathways. Most studies have shown that high expression of CXCR4 is closely related to a poor prognosis. Therefore, CXCR4 plays an important role in the occurrence and development of GC. However, whether the expression of CXCR4 is correlated with the TMB, CYT, immune infiltration, and tumor purity of GC is still unknown.

In this present study, we showed that CXCR4 is overexpressed in GC tumor tissue, with a significant correlation between the CXCR4 levels and the survival rate, consistent with previous studies. Moreover, we found that a high CXCR4 expression in patients was correlated with a more advanced clinical stage, which is an extremely important prognostic factor for tumor progression [31, 32]. For the T stage, we found a statistical difference only in cohort 2 and not in cohort 1. However, in cohort 1, the total number of patients with a T3 and T4 stage tumor in the CXCR4‐H group was higher than that in the CXCR4‐L group. These results are consistent with previous studies reporting that in patients with high CXCR4 expression, both the depth of tumor invasion and the amount of distant metastasis are higher than those in patients with a low expression [33, 34]. The difference between the cohorts may be explained by the absence of patients with a T1 stage tumor in cohort 1, which may limit the power of the statistical analysis. We also found no significant difference between the two groups regarding the M stage, probably because of the small number of M1 patients available. In terms of N stage, we found a significant difference in cohort 1, but no significant progressiveness. Collectively, our results indicate that CXCR4 may have an effect on local and distant invasion in GC. More patient data should be collected to validate our analysis.

We also focused our attention on another important characteristic, tumor immune infiltration. The effect of immune infiltration cells on the prognosis of GC is still controversial [35]. In the present study, genes involved in the immune and the inflammation response were enriched in the fraction of genes showing a differential expression between the CXCR4‐H and CXCR4‐L groups. CXCR4 expression was highly correlated with T cells, macrophages, dendritic cells, and other immune cells. Moreover, we verified this correlation by observing a similar correlation between CXCR4 expression and immune gene markers. Then, we calculated the immune infiltration levels of each patient by CIBERSORT, and we found that, in both cohorts, in the CXCR4‐H group, the fraction of B cells and CD8^+^ T cells was higher, while the fraction of CD4^+^ T cells was lower compared to the CXCR4‐L group. A recent study showed that a high number of CD8^+^ T cells positively correlate with poor OS [36]. Another study reported that an increased intratumor CD8^+^ lymphocyte ratio is correlated with a decreased OS [37]. Moreover, an increased CD4^+^ T‐cell number represents a favorable prognostic factor [38]. We also found that in cohort 1, the number of monocytes in the CXCR4‐H group was lower than that in the CXCR4‐L group, while in cohort 2, the number of NK cells was lower. These results are consistent with previous studies showing an antitumor role for monocytes and NK cells in the tumor microenvironment. These two cell types also have a positive effect on GC prognosis [39, 40].

We then investigated whether there was a difference in the extent of immune cell infiltration and SCNA of the CXCR4 gene. We observed that the majority of changes occurred in concomitance with arm‐level deletion and arm‐level gain. When CXCR4 had arm‐level deletion or arm‐level gain, the mRNA expression level of both groups decreased, but only the decrease in the arm‐level deletion group was significant compared with that of the normal group. There was no significant difference in the gain group. As shown in Fig. 5, the expression of CXCR4 was positively correlated with B cells, CD4, CD8, macrophages, neutrophils, and dendritic cells, indicating that when the expression of CXCR4 was greatly reduced, the infiltration degree of these immune cells would also be reduced, so the immune cells in the arm‐level deletion group all decreased compared with the normal group. The decrease in the arm‐level gain group was not statistically significant. Thus, the decrease in the number of immune cells was only different between macrophages and dendritic cells, but the difference was not obvious. Therefore, it can be observed that when copy number variation of CXCR4 occurs, especially when arm‐level deletion occurs, the expression of CXCR4 will be decreased, thus affecting the infiltration of immune cells.

Tumor purity is another aspect that has been thoroughly investigated in recent years. Many studies showed that tumor purity could become an important parameter in tumor research [18, 41]. By calculating the tumor purity in our cohorts, we observed that in the CXCR4‐H group, the tumor stromal score and the immune score were higher than in the CXCR4‐L group, while the tumor purity was lower than that in the CXCR4‐L group. Previous studies showed that patients with a high tumor purity have a better prognosis. A high stromal and immune score in tumor tissues are correlated with a lack of infiltration and function of chemotherapy drugs [42, 43]. Consistently, our results showed that patients with high CXCR4 expression have a high IC50 response to three antitumor drugs.

The TMB value is generally expressed in terms of the total number of nonsynonymous mutations or the number of mutations per Mb (1 Mb base). Our immune system eliminates abnormal cells; the idea behind tumor immunotherapy is to boost and strengthen the body's immune system with various methods to kill the tumor cells. In this context, the higher the TMB is, the greater the difference between tumor cells and normal cells. Thus, high TMB values reflect a high predisposition to be the target of antitumor immunity. Consistent with our survival and drug sensitivity results, numerous studies confirmed that immunotherapy is more efficient in patients with high TMB than in patients with low TMB [43, 44]. In our study, patients with a high expression of CXCR4 had a lower TMB than patients with a low CXCR4 expression, suggesting that patients with high expression of CXCR4 may be resistant to immunotherapy, thus explaining the worse prognosis.

The CYT is calculated based on the GZMA and PRF1 levels. CYT is dramatically upregulated upon CD8^+^ T‐cell activation and during productive clinical responses to anti‐CTLA‐4 and anti‐PD‐L1 immunotherapies [20]. Previous studies [45, 46] showed that in glioma and colorectal cancers, an increased CYT is correlated with an improved prognosis [47]. However, the relevance of CYT in GC is unknown. In our study, CYT and the CD8^+^ T‐cell fraction were higher in the CXCR4‐H group. Thus, it seems that in GC, CYT is higher in the CXCR4 groups characterized by a worse survival rate, in contrast with previous studies. We think that the CYT with worse survival could be high in this group for several reasons. Patients with a high CXCR4 expression have a lower tumor purity, which can lead to poor survival. An increase in CD8^+^ T‐cell infiltration could also affect the prognosis. Moreover, whether a high CYT can lead to an improved survival also in GC patients is still unknown. More studies on the prognostic role of CYT in GC patients are needed. Our results showed that despite a high CYT in the CXCR4‐H group, the prognosis of these patients is lower than that of patients in the CXCR4‐L group for a variety of reasons.

Drug sensitivity is an important prognostic factor for GC patients. Previous studies indicated that CXCR4 overexpression could protect tumor cells from chemotherapy drugs [48, 49]. The 17‐agg drug is an HSP90 inhibitor. Recent studies showed that in lung adenocarcinoma and breast cancer, 17‐aag can inhibit the growth and induce the apoptosis of tumor cells [50, 51, 52]. Other studies showed that 17‐agg combined with platinum can inhibit the growth of GC cells [53]. Trametinib, a specific MEK inhibitor, is mainly used for the treatment of adult patients with unresectable melanoma or metastatic melanoma who carry either the BRAF V600E or the V600K mutations [54]. Its use for the treatment of advanced non‐small‐cell lung cancer with the BRAF V600E mutation has also been reported [55]. Some studies also showed that trametinib can be used to induce apoptosis of GC cells in combination with other drugs. However, 17‐agg and trametinib are rarely used in the conventional clinical treatment of GC. Docetaxel has been shown to be more effective for GC treatment; various studies suggested that a combination of docetaxel and other drugs can be used to prolong the survival in patients with advanced GC [56, 57, 58]. In the present study, we discovered that the CXCR4 levels can affect the resistance of cancer cells to three drugs. When CXCR4 expression is increased, the resistance to the three drugs also increases. These results could guide physicians in clinical treatment selection, especially for docetaxel. Patients with a low expression of CXCR4 may benefit from a docetaxel treatment, although further verification with more samples is needed.

In summary, CXCR4 is an important independent factor to evaluate the prognosis of GC patients. We found a statistically significant correlation between CXCR4 and TMB, CYT, tumor purity, tumor immune infiltration, drug sensitivity, and other clinical parameters playing an important role in GC insurgence and development.

## Conflict of interest

The authors declare no conflict of interest.

## Author contributions

YL and LL conceived and designed the project. H‐CW and BS acquired the data. YL and H‐CW analyzed and interpreted the data. J‐SW completed the statistical calculations. YL complete the original draft preparation. J‐SW and LL finished the review and editing.

## Supporting information


**Table S1.** GO‐BP and KEGG analysis of the two cohorts.
**Table S2.** Immune gene markers.
**Table S3.** GDSC analysis.

## References

[1] Kamangar F (2006) Patterns of cancer incidence, mortality, and prevalence across five continents: defining priorities to reduce cancer disparities in different geographic regions of the world. J Clin Oncol 24, 2137–2150. 16682732 10.1200/JCO.2005.05.2308

[2] Ferlay J , Soerjomataram I , Dikshit R , Eser S , Mathers C , Rebelo M , Parkin DM , Forman D and Bray F (2015) Cancer incidence and mortality worldwide: sources, methods and major patterns in GLOBOCAN 2012. Int J Cancer 136, E359–E386. 25220842 10.1002/ijc.29210

[3] Jung KW , Won YJ , Kong HJ , Oh CM , Shin A and Lee JS (2013) Survival of Korean adult cancer patients by stage at diagnosis, 2006–2010: National Cancer Registry Study. Cancer Res Treat 45, 162–171. 24155674 10.4143/crt.2013.45.3.162 PMC3804727

[4] Cho JY (2013) Molecular diagnosis for personalized target therapy in gastric cancer. J Gastric Cancer 13, 129–135. 24156032 10.5230/jgc.2013.13.3.129 PMC3804671

[5] Derlin T and Hueper K (2018) CXCR4‐targeted therapy in breast cancer. Lancet Oncol 19, e370. 30102220 10.1016/S1470-2045(18)30480-7

[6] Gao Y , Li C , Nie M , Lu Y , Lin S , Yuan P and Sun X (2014) CXCR4 as a novel predictive biomarker for metastasis and poor prognosis in colorectal cancer. Tumor Biol 35, 4171–4175. 10.1007/s13277-013-1545-x 24395653

[7] Xiang Z , Zhou ZJ , Xia GK , Zhang XH , Wei ZW , Zhu JT , Yu J , Chen W , He Y , Schwarz RE *et al* (2017) A positive crosstalk between CXCR4 and CXCR2 promotes gastric cancer metastasis. Oncogene 36, 5122–5133. 28481874 10.1038/onc.2017.108

[8] Domanska UM , Kruizinga RC , Nagengast WB , Timmer‐Bosscha H , Huls G , de Vries EGE and Walenkamp AME (2013) A review on CXCR4/CXCL12 axis in oncology: no place to hide. Eur J Cancer 49, 219–230. 22683307 10.1016/j.ejca.2012.05.005

[9] Guo F , Wang Y , Liu J , Mok SC , Xue F and Zhang W (2015) CXCL12/CXCR4: a symbiotic bridge linking cancer cells and their stromal neighbors in oncogenic communication networks. Oncogene 35, 816. 25961926 10.1038/onc.2015.139

[10] Abe P , Wüst HM , Arnold SJ , van de Pavert SA and Stumm R (2018) CXCL12‐mediated feedback from granule neurons regulates generation and positioning of new neurons in the dentate gyrus. Glia 66, 1566–1576. 29537098 10.1002/glia.23324

[11] Bao Y , Wang Z , Liu B , Lu X , Xiong Y , Shi J , Li P , Chen J , Zhang Z , Chen M *et al* (2019) A feed‐forward loop between nuclear translocation of CXCR4 and HIF‐1α promotes renal cell carcinoma metastasis. Oncogene 38, 881–895. 30177838 10.1038/s41388-018-0452-4 PMC6367212

[12] Lin XL , Xu Q , Tang L , Sun L and Xiao XY (2017) Regorafenib inhibited gastric cancer cells growth and invasion via CXCR4 activated Wnt pathway. PLoS ONE 12, e0177335. 28489887 10.1371/journal.pone.0177335 PMC5425213

[13] Lee HJ , Kim SW , Kim HY , Li S , Yun HJ , Song KS , Kim S and Jo DY (2009) Chemokine receptor CXCR4 expression, function, and clinical implications in gastric cancer. Int J Oncol 34, 473–480. 19148483

[14] Fontanella R , Pelagalli A , Nardelli A , D'Alterio C , Ierano C , Cerchia L , Lucarelli E , Scala S and Zannetti A (2016) A novel antagonist of CXCR4 prevents bone marrow‐derived mesenchymal stem cell‐mediated osteosarcoma and hepatocellular carcinoma cell migration and invasion. Cancer Lett 370, 100–107. 26517945 10.1016/j.canlet.2015.10.018

[15] Zeng D , Zhou R , Yu Y , Luo Y , Zhang J , Sun H , Bin J , Liao Y , Rao J , Zhang Y *et al* (2018) Gene expression profiles for a prognostic immunoscore in gastric cancer. Br J Surg 105, 1338–1348. 29691839 10.1002/bjs.10871 PMC6099214

[16] Hellmann MD , Callahan MK , Awad MM , Calvo E , Ascierto PA , Atmaca A , Rizvi NA , Hirsch FR , Selvaggi G , Szustakowski JD *et al* (2018) Tumor mutational burden and efficacy of nivolumab monotherapy and in combination with ipilimumab in small‐cell lung cancer. Cancer Cell 33, 853–861.e4. 29731394 10.1016/j.ccell.2018.04.001 PMC6750707

[17] Chu Y , Teng M and Wang Y (2018) Modeling and correct the GC bias of tumor and normal WGS data for SCNA based tumor subclonal population inferring. BMC Bioinformatics 19, 112. 29671389 10.1186/s12859-018-2099-0 PMC5907144

[18] Rhee J‐K , Jung YC , Kim KR , Yoo J , Kim J , Lee Y‐J , Ko YH , Lee HH , Cho BC and Kim T‐M (2018) Impact of tumor purity on immune gene expression and clustering analyses across multiple cancer types. Cancer Immunol Res 6, 87–97. 29141981 10.1158/2326-6066.CIR-17-0201

[19] Junttila MR and de Sauvage FJ (2013) Influence of tumour micro‐environment heterogeneity on therapeutic response. Nature 501, 346–354. 24048067 10.1038/nature12626

[20] Rooney MS , Shukla SA , Wu CJ , Getz G and Hacohen N (2015) Molecular and genetic properties of tumors associated with local immune cytolytic activity. Cell 160, 48–61. 25594174 10.1016/j.cell.2014.12.033 PMC4856474

[21] Cristescu R , Lee J , Nebozhyn M , Kim KM , Ting JC , Wong SS , Liu J , Yue YG , Wang J , Yu K *et al* (2015) Molecular analysis of gastric cancer identifies subtypes associated with distinct clinical outcomes. Nat Med 21, 449–456. 25894828 10.1038/nm.3850

[22] Ooi CH , Ivanova T , Wu J , Lee M , Tan IB , Tao J , Ward L , Koo JH , Gopalakrishnan V , Zhu Y *et al* (2009) Oncogenic pathway combina‐tions predict clinical prognosis in gastric cancer. PLoS Genet 5, e1000676. 19798449 10.1371/journal.pgen.1000676 PMC2748685

[23] Qian Z , Zhu G , Tang L , Wang M , Zhang L , Fu J , Huang C , Fan S , Sun Y , Lv J *et al* (2014) Whole genome gene copy number profiling of gastric cancer identifies PAK1 and KRAS gene amplification as therapy targets. Genes Chromosomes Cancer 53, 883–894. 24935174 10.1002/gcc.22196

[24] Lei Z , Tan IB , Das K , Deng N , Zouridis H , Pattison S , Chua C , Feng Z , Guan YK , Ooi CH *et al* (2013) Identification of molecular subtypes of gastric cancer with different responses to PI3‐kinase inhibitors and 5‐fluorouracil. Gastroenterology 145, 554–565. 23684942 10.1053/j.gastro.2013.05.010

[25] Irizarry AR (2003) Exploration, normalization, and summaries of high density oligonucleotide array probe level data. Biostatistics 4, 249–264. 12925520 10.1093/biostatistics/4.2.249

[26] Huang DW , Sherman BT and Lempicki RA (2009) Systematic and integrative analysis of large gene lists using DAVID bioinformatics resources. Nat Protoc 4, 44–57. 19131956 10.1038/nprot.2008.211

[27] Pan J‐H , Zhou H , Cooper L , Huang J‐L , Zhu S‐B , Zhao X‐X , Ding H , Pan Y‐L and Rong L (2019) LAYN is a prognostic biomarker and correlated with immune infiltrates in gastric and colon cancers. Front Immunol 10, 6. 30761122 10.3389/fimmu.2019.00006 PMC6362421

[28] Chen B , Khodadoust MS , Liu CL , Newman AM and Alizadeh AA (2018) Profiling tumor infiltrating immune cells with CIBERSORT In Cancer Systems Biology: Methods and Protocols (von Stechow L, ed.), pp. 243–259. Springer, New York, NY. 10.1007/978-1-4939-7493-1_12 PMC5895181 29344893

[29] Yoshihara K , Shahmoradgoli M , Martínez E , Vegesna R , Kim H , Torres‐Garcia W , Treviño V , Shen H , Laird PW , Levine DA *et al* (2013) Inferring tumour purity and stromal and immune cell admixture from expression data. Nat Commun 4, 2612. 24113773 10.1038/ncomms3612 PMC3826632

[30] Yang W , Soares J , Greninger P , Edelman EJ , Lightfoot H , Forbes S , Bindal N , Beare D , Smith JA , Thompson IR *et al* (2013) Genomics of Drug Sensitivity in Cancer (GDSC): a resource for therapeutic biomarker discovery in cancer cells. Nucleic Acids Res 41, D955–D961. 23180760 10.1093/nar/gks1111 PMC3531057

[31] Choi YY , Jang E , Seo WJ , Son T , Kim H‐I , Kim H , Hyung WJ , Huh Y‐M , Noh SH and Cheong J‐H (2018) Modification of the TNM staging system for stage II/III gastric cancer based on a prognostic single patient classifier algorithm. J Gastric Cancer 18, 142–151. 29984064 10.5230/jgc.2018.18.e14 PMC6026714

[32] Boeker M , França F , Bronsert P and Schulz S (2016) TNM‐O: ontology support for staging of malignant tumours. J Biomed Semantics 7, 64. 27842575 10.1186/s13326-016-0106-9 PMC5109740

[33] Yamada S , Shimada M , Utsunomiya T , Morine Y , Imura S , Ikemoto T , Mori H , Arakawa Y , Kanamoto M , Iwahashi S *et al* (2014) CXC receptor 4 and stromal cell–derived factor 1 in primary tumors and liver metastases of colorectal cancer. J Surg Res 187, 107–112. 24238971 10.1016/j.jss.2013.10.030

[34] Liu Y , Ren CC , Yang L , Xu YM and Chen YN (2019) Role of CXCL12‐CXCR4 axis in ovarian cancer metastasis and CXCL12‐CXCR4 blockade with AMD3100 suppresses tumor cell migration and invasion in vitro. J Cell Physiol 234, 3897–3909. 30191987 10.1002/jcp.27163

[35] Lazar DC , Avram MF , Romosan I , Cornianu M , Taban S and Goldis A (2018) Prognostic significance of tumor immune microenvironment and immunotherapy: novel insights and future perspectives in gastric cancer. World J Gastroenterol 24, 3583–3616. 30166856 10.3748/wjg.v24.i32.3583 PMC6113718

[36] Thompson ED , Zahurak M , Murphy A , Cornish T , Cuka N , Abdelfatah E , Yang S , Duncan M , Ahuja N , Taube JM *et al* (2017) Patterns of PD‐L1 expression and CD8 T cell infiltration in gastric adenocarcinomas and associated immune stroma. Gut 66, 794–801. 26801886 10.1136/gutjnl-2015-310839 PMC4958028

[37] Shen Z , Zhou S , Wang Y , Li RL , Zhong C , Liang C and Sun Y (2010) Higher intratumoral infiltrated Foxp3+ Treg numbers and Foxp3+/CD8+ ratio are associated with adverse prognosis in resectable gastric cancer. J Cancer Res Clin Oncol 136, 1585–1595. 20221835 10.1007/s00432-010-0816-9 PMC11828043

[38] Ubukata H , Motohashi G , Tabuchi T , Nagata H , Konishi S and Tabuchi T (2010) Evaluations of interferon‐γ/interleukin‐4 ratio and neutrophil/lymphocyte ratio as prognostic indicators in gastric cancer patients. J Surg Oncol 102, 742–747. 20872813 10.1002/jso.21725

[39] Peng LS , Zhang JY , Teng YS , Zhao YL , Wang TT , Mao FY , Lv YP , Cheng P , Li WH , Chen N *et al* (2017) Tumor‐associated monocytes/macrophages impair NK‐cell function via TGFbeta1 in human gastric cancer. Cancer Immunol Res 5, 248–256. 28148545 10.1158/2326-6066.CIR-16-0152

[40] Betten Å , Bylund J , Cristophe T , Boulay F , Romero A , Hellstrand K and Dahlgren C (2001) A proinflammatory peptide from Helicobacter pylori activates monocytes to induce lymphocyte dysfunction and apoptosis. J Clin Invest 108, 1221–1228. 11602630 10.1172/JCI13430 PMC209532

[41] Aran D , Sirota M and Butte AJ (2015) Systematic pan‐cancer analysis of tumour purity. Nat Commun 6, 8971. 26634437 10.1038/ncomms9971 PMC4671203

[42] Park JH , Richards CH , McMillan DC , Horgan PG and Roxburgh CS (2014) The relationship between tumour stroma percentage, the tumour microenvironment and survival in patients with primary operable colorectal cancer. Ann Oncol 25, 644–651. 24458470 10.1093/annonc/mdt593 PMC4433525

[43] Mao Y , Feng Q , Zheng P , Yang L , Liu T , Xu Y , Zhu D , Chang W , Ji M , Ren L *et al* (2018) Low tumor purity is associated with poor prognosis, heavy mutation burden, and intense immune phenotype in colon cancer. Cancer Manag Res 10, 3569–3577. 30271205 10.2147/CMAR.S171855 PMC6149864

[44] Maleki Vareki S (2018) High and low mutational burden tumors versus immunologically hot and cold tumors and response to immune checkpoint inhibitors. J Immunother Cancer 6, 157. 30587233 10.1186/s40425-018-0479-7 PMC6307306

[45] Zaravinos A , Roufas C , Nagara M , de Lucas Moreno B , Oblovatskaya M , Efstathiades C , Dimopoulos C and Ayiomamitis GD (2019) Cytolytic activity correlates with the mutational burden and deregulated expression of immune checkpoints in colorectal cancer. J Exp Clin Cancer Res 38, 364. 31429779 10.1186/s13046-019-1372-z PMC6701076

[46] Roufas C , Chasiotis D , Makris A , Efstathiades C , Dimopoulos C and Zaravinos A (2018) The expression and prognostic impact of immune cytolytic activity‐related markers in human malignancies: a comprehensive meta‐analysis. Front Oncol 8, 27. 29515971 10.3389/fonc.2018.00027 PMC5826382

[47] Narayanan S , Kawaguchi T , Yan L , Peng X , Qi Q and Takabe K (2018) Cytolytic activity score to assess anticancer immunity in colorectal cancer. Ann Surg Oncol 25, 2323–2331. 29770915 10.1245/s10434-018-6506-6 PMC6237091

[48] Lecavalier‐Barsoum M , Chaudary N , Han K , Pintilie M , Hill RP and Milosevic M (2019) Targeting CXCL12/CXCR4 and myeloid cells to improve the therapeutic ratio in patient‐derived cervical cancer models treated with radio‐chemotherapy. Br J Cancer 121, 249–256. 31239542 10.1038/s41416-019-0497-3 PMC6738100

[49] Lecavalier‐Barsoum M , Chaudary N , Han K , Koritzinsky M , Hill R and Milosevic M (2018) Targeting the CXCL12/CXCR4 pathway and myeloid cells to improve radiation treatment of locally advanced cervical cancer. Int J Cancer 143, 1017–1028. 29417588 10.1002/ijc.31297

[50] Tsai MS , Weng SH , Chen HJ , Chiu YF and Huang YC (2012) Inhibition of p38 MAPK‐dependent excision repair cross‐complementing 1 expression decreases the DNA repair capacity to sensitize lung cancer cells to etoposide. Mol Cancer Ther 11, 561–571. 22053010 10.1158/1535-7163.MCT-11-0684

[51] Pimienta G , Herbert KM and Regan L (2011) A compound that inhibits the HOP–Hsp90 complex formation and has unique killing effects in breast cancer cell lines. Mol Pharm 8, 2252–2261. 21882818 10.1021/mp200346y

[52] Zuo Y , Wang J , Liao F , Yan X , Li J , Huang L and Liu F (2018) Inhibition of heat shock protein 90 by 17‐AAG reduces inflammation via P2X7 receptor/NLRP3 inflammasome pathway and increases neurogenesis after subarachnoid hemorrhage in mice. Front Mol Neurosci 11, 401. 30459553 10.3389/fnmol.2018.00401 PMC6232389

[53] Chen MN , Guo W , Jing hong XU and Zhao JM (2013) Effect of 17‐AAG combined with cisplatin on proliferation and apoptosis of human gastric cancer cell lines SGC‐7901. J Kunming Med Univ 34, 54–57.

[54] Long GV , Stroyakovskiy D , Gogas H , Levchenko E , Braud FD , Larkin J , Garbe C , Jouary T , Hauschild A , Grob J‐J *et al* (2015) Dabrafenib and trametinib versus dabrafenib and placebo for Val600 BRAF‐mutant melanoma: a multicentre, double‐blind, phase 3 randomised controlled trial. Lancet 386, 444–451. 26037941 10.1016/S0140-6736(15)60898-4

[55] Planchard D , Smit EF , Hjm G , Mazieres J , Besse B , Helland Å , Giannone V , D'Amelio AM , Zhang P , Mookerjee B *et al* (2017) Dabrafenib plus trametinib in patients with previously untreated BRAFV600E‐mutant metastatic non‐small‐cell lung cancer: an open‐label, phase 2 trial. Lancet Oncol 18, 1307–1316. 28919011 10.1016/S1470-2045(17)30679-4

[56] Thuss‐Patience PC , Hofheinz RD , Arnold D , Florschutz A , Daum S , Kretzschmar A , Mantovani‐Löffler L , Bichev D , Breithaupt K , Kneba M *et al* (2012) Perioperative chemotherapy with docetaxel, cisplatin and capecitabine (DCX) in gastro‐oesophageal adenocarcinoma: a phase II study of the Arbeitsgemeinschaft Internistische Onkologie (AIO){dagger}. Ann Oncol 23, 2827–2834. 22734012 10.1093/annonc/mds129

[57] Al‐Batran S‐E , Hofheinz RD , Pauligk C , Kopp H‐G , Haag GM , Luley KB , Meiler J , Homann N , Lorenzen S , Schmalenberg H *et al* (2016) Histopathological regression after neoadjuvant docetaxel, oxaliplatin, fluorouracil, and leucovorin versus epirubicin, cisplatin, and fluorouracil or capecitabine in patients with resectable gastric or gastro‐oesophageal junction adenocarcinoma (FLOT4‐AIO): results from the phase 2 part of a multicentre, open‐label, randomised phase 2/3 trial. Lancet Oncol 17, 1697–1708. 27776843 10.1016/S1470-2045(16)30531-9

[58] Lorenzen S , Pauligk C , Homann N , Schmalenberg H , Jäger E and Al‐Batran SE (2013) Feasibility of perioperative chemotherapy with infusional 5‐FU, leucovorin, and oxaliplatin with (FLOT) or without (FLO) docetaxel in elderly patients with locally advanced esophagogastric cancer. Br J Cancer 108, 519–526. 23322206 10.1038/bjc.2012.588 PMC3593547

