# Supplementary material for: Chemokine receptor 4 expression is correlated with the occurrence and prognosis of gastric cancer
Source: FEBS Open Bio. 2020 May 7;10(6):1149–61. doi: 10.1002/2211-5463.12864 (PMC7262922; doi:10.1002/2211-5463.12864)
Supplement: Supplementary file 1 — Table S1. GO‐BP and KEGG analysis of the two cohorts. Table S2. Immune gene markers. Table S3. GDSC analysis. [file FEB4-10-1149-s001.docx]

**Table S1: GO-BP and KEGG analysis of the two cohorts**

| **Cohort 1** |  |  |  |
| --- | --- | --- | --- |
| Term | Count | P value | FDR |
| GO:0006955~immune response | 86 | 4.48E-36 | 8.10E-33 |
| GO:0006954~inflammatory response | 68 | 4.96E-25 | 8.96E-22 |
| GO:0007155~cell adhesion | 67 | 1.20E-19 | 2.16E-16 |
| GO:0050853~B cell receptor signaling pathway | 23 | 3.59E-17 | 6.49E-14 |
| GO:0030198~extracellular matrix organization | 38 | 3.31E-15 | 6.02E-12 |
| GO:0002250~adaptive immune response | 33 | 4.57E-15 | 8.22E-12 |
| GO:0045087~innate immune response | 57 | 8.35E-15 | 1.50E-11 |
| GO:0006935~chemotaxis | 29 | 4.82E-14 | 8.72E-11 |
| GO:0050776~regulation of immune response | 34 | 1.81E-13 | 3.27E-10 |
| GO:0070098~chemokine-mediated signaling pathway | 21 | 3.45E-12 | 6.23E-09 |
|  |  |  |  |
| **Cohort 2** |  |  |  |
| Term | Count | PValue | FDR |
| GO:0008544~epidermis development | 36 | 5.98E-18 | 1.10E-14 |
| GO:0018149~peptide cross-linking | 27 | 9.23E-17 | 2.00E-13 |
| GO:0002250~adaptive immune response | 46 | 1.15E-16 | 2.00E-13 |
| GO:0030216~keratinocyte differentiation | 32 | 7.49E-16 | 1.43E-12 |
| GO:0031424~keratinization | 24 | 5.86E-14 | 1.08E-10 |
| GO:0006955~immune response | 73 | 3.18E-11 | 5.85E-08 |
| GO:0045087~innate immune response | 72 | 2.24E-10 | 4.12E-07 |
| GO:0006954~inflammatory response | 64 | 1.79E-09 | 3.30E-06 |
| GO:0042102~positive regulation of T cell proliferation | 21 | 6.60E-09 | 1.22E-05 |
| GO:0050830~defense response to Gram-positive bacterium | 25 | 8.92E-09 | 1.64E-05 |

**Table S1 GO-BP and KEGG analysis of the two cohorts.** **The first column shows the numbers and names of the enrichment results; the second column shows the number of the genes enriched in this term; the third column shows the P value; the fourth column shows the FDR**

**Table S2: Immune gene markers**

| Description | Gene markers |
| --- | --- |
| Th1 | CD38, STAT1, CTLA4, IL12RB2, LTA, STAT4, TBX21 |
| Th2 | CXCR6, GATA3, IL13, LAIR2, PMCH, SMAD2, STAT6，IL26 |
| Treg | FOXP3, CCR8, STAT5B, TGFβ (TGFB1), CD3E, CD4, CD25(IL2RA), |
| Tfh (follicular helper) | BCL6, CXCL13, MAF, PDCD1，IL21 |
| Tgd (gamma delta) | CD160, FEZ1, TARP |
| B cells | BLK, CD19, CD79A CR2, HLA-DOB, MS4A1, TNFRSF17, MME |
| M1 Macrophage | CD68, CD86, CD80, CD11b(ITGAM), CD282(TLR2), CD284(TLR4) |
| M2 Macrophage | CD163, VSIG4, MS4A4A, CCR7, CD206(MRC1), CD200R(CD200R1), |
| Neutrophils | CD66b (CEACAM8), CD11b (ITGAM), CCR7, CSF3R, FCGR3A |
| Natural killer cells | BCL2, FCGR3A, NCR1, NCAM1 |
| Dendritic cell | HLA-DPB1, HLA-DQB1, HLA-DRA, HLA-DPA1, BDCA-1(CD1C), BDCA-4(NRP1), CD11c (ITGAX), CD83, CD1A, CD1B, CD1E, CCL13, CCL17, CCL22, CD209, HSD11B1, EBI3, F13A1 |

**Table S2 Immune gene markers. The genes selected for the validation of the correlation between CXCR4 expression and immune infiltration cells.**

**Table S3: GDSC analysis**

| Gene | Drug ID | Drug names | Cor | P value |
| --- | --- | --- | --- | --- |
| CXCR4 | 1026 | 17-AAG | 0.391 | 0.001664 |
| CXCR4 | 1372 | Trametinib | 0.338 | 0.004489 |
| CXCR4 | 1007 | Docetaxel | 0.293 | 0.009752 |
| CXCR4 | 1060 | PD-0325901 | 0.272 | 0.01372 |
| CXCR4 | 1526 | RDEA119 | 0.271 | 0.01394 |
| CXCR4 | 1014 | RDEA119 | 0.265 | 0.015328 |
| CXCR4 | 1378 | Bleomycin (50 uM) | 0.243 | 0.021515 |
| CXCR4 | 1498 | selumetinib | 0.229 | 0.026498 |
| CXCR4 | 51 | Dasatinib | 0.172 | 0.058335 |
| CXCR4 | 173 | FH535 | 0.157 | 0.070689 |
| CXCR4 | 1008 | Methotrexate | -0.472 | 0.074048 |
| CXCR4 | 275 | I-BET-762 | -0.465 | 0.080755 |
| CXCR4 | 290 | KIN001-260 | -0.461 | 0.084804 |
| CXCR4 | 257 | NPK76-II-72-1 | -0.46 | 0.085841 |
| CXCR4 | 286 | KIN001-236 | -0.459 | 0.086889 |
| CXCR4 | 265 | Tubastatin A | -0.45 | 0.09679 |
| CXCR4 | 1011 | Navitoclax | -0.444 | 0.103878 |
| CXCR4 | 226 | GSK1070916 | -0.44 | 0.108828 |
| CXCR4 | 303 | PIK-93 | -0.429 | 0.123405 |
| CXCR4 | 301 | PHA-793887 | -0.429 | 0.123405 |

**Table S3 GDSC analysis. The first column shows the name of the gene; the second column shows the drug ID; the third column shows the drug names; the fourth column shows the correlation between CXCR4 and drugs; the fifth column shows the P value of the correlation. Only the top 10 positive and negative drugs are shown, respectively.**
